# Metabolic changes in mTOR pathway-associated cortical malformation

**DOI:** 10.3389/fnins.2026.1726799

**Published:** 2026-02-18

**Authors:** Aditi Biswas, Philip H. Iffland

**Affiliations:** 1 Department of Psychiatry, University of Maryland School of Medicine, Baltimore, MD, United States; 2 Institute for Genome Sciences, University of Maryland School of Medicine, Baltimore, MD, United States; 3 Department of Neurology, University of Maryland School of Medicine, Baltimore, MD, United States

**Keywords:** brain development, developmental delay, epilepsy, intellectual disability, neurodevelopmental disorders, seizures, metabolism

## Abstract

Malformations of cortical development (MCD) are a common cause of epilepsy, autism spectrum disorder (ASD), and intellectual disability (ID). A unifying mechanistic etiology for epilepsy, ASD, and ID has not been found due, in part, to the heterogeneity of MCD subtypes. However, changes in brain metabolism within MCD have emerged as a possible convergent mechanism across MCD that may have therapeutic potential. While there have been efforts to comprehensively describe known metabolic changes in other neurodevelopmental disorders, no such effort exists for MCD associated with mTOR pathway gene mutations (the most common cause of MCD; mTORopathies’). In this review, we detail finding related to mTORopathies that relate to dysfunctional brain metabolism including abnormal changes in macromolecule processing and mitochondrial metabolism. Further, we discuss cellular and molecular metabolic processes that may serve as key pathways in the development of MCD *in utero* and that may ultimately produce common mTORopathy phenotypes and sustain abnormal brain activity post-development.

## Introduction

Efforts to identify and characterize malformations of cortical development (MCD) have largely focused on defining the genetic, developmental, and morphological changes that result in cortical malformations and associated phenotypes. Compared to other mammalian cortices, the human cortex develops for a longer period with a corresponding increase in overall brain seize brain size. This expansion is due to more rounds of cell division and migration, as well as refinement and pruning of neural circuits that continue for years after birth. Key drivers of cortical development are complex metabolic processes that change over the developmental timespan and continue to persist long after the brain has fully developed. Thus, metabolic changes within MCD have the potential to impact individuals across their entire lifespan (Troha and Ayres, 2020; Iwata et al., 2023; Yang et al., 2024) and may contribute directly to epileptogenesis and/or be a secondary effect of recurrent seizures. Thus, investigating the changes in cellular metabolism in mTORopathies and other, similar, MCD is crucial to determining whether what role metabolic changes play in the pathogenesis of these disorders and whether these changes can be targets for therapy. Key to understanding metabolic dysregulation in MCD are the processing of macromolecules (which serve as energy substrates) and mitochondrial function (which affects oxidative metabolism).

## Malformations of cortical development and the mTOR pathway

**FIGURE 1:**
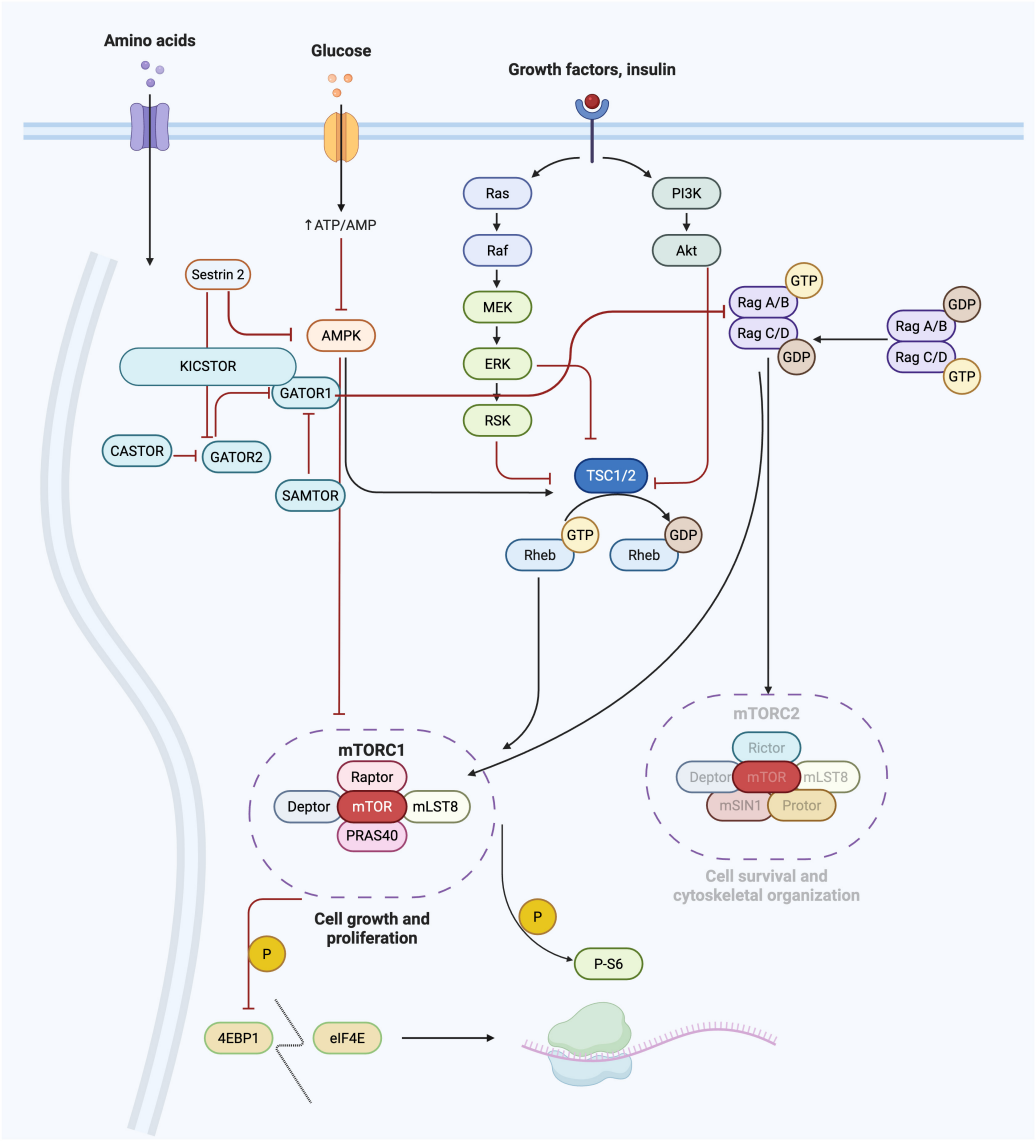
mTOR pathway signaling cascade. The major components of the three primary arms of the mTOR pathway are shown including the amino acid, glucose, and growth factor (canonical) arms of the pathway. These nutrient signaling cues from the cellular environments ultimately feed into the master kinase complexes mTORC1 and mTORC2. Metabolic functions largely impact, and are impacted by, mTORC1.

The PI3K/AKT/mTOR (mTOR) pathway is a key regulator of cell cycle, protein biogenesis, autophagy, and mitochondrial function (Laplante and Sabatini, 2009). mTOR itself is the catalytic unit of two signaling complexes, mTORC1 and mTORC2 and, of the two, mTORC1 has a more defined role in the regulation of cellular metabolism and nutrient-sensing (Figure 1). Indeed, mTORC1 kinase activity is modulated by amino acids, lipids, and glucose (Goul et al., 2023). In turn, mTOR pathway activity facilitates downstream cellular metabolic pathways that drive cell proliferation, differentiation, decreased autophagy, and lysosome biogenesis during brain development. Indeed, the mTOR pathway plays a key role in cortical development and changes in signaling can result in MCD (Andrews et al., 2020).

MCD are defined by cytoarchitectural and gross structural changes in the cerebral cortex. MCD are highly heterogenous in genotype and phenotype and the spatial and temporal context the malformation is occurring in can inform the size and location of the malformation. For example, histological abnormalities associated with MCD include cortical dyslamination, loss of differentiation between gray and white matter, cytomegalic dysmorphic neurons, and heterotopic neurons in white matter (Guerrini and Dobyns, 2014)- though the exact combination of these changes can vary with MCD subtype. Individuals with MCD can have seizures, developmental delays, intellectual disability (ID), and/or Autism Spectrum Disorder (ASD) (Pang et al., 2008). There are many classification systems for MCD, but there are three major subtypes based on cellular abnormalities: (1) MCD due to deficits in cell proliferation and survival, (2) MCD resulting from migration deficits, and (3) MCD occurring from post-migration differentiation and connectivity deficits (Ossola and Kalebic, 2022). The most common MCD subtype are caused by genetic variants in mTOR pathway subunits that ultimately result in mTOR pathway hyperactivation and dysregulation of downstream processes under the control of mTOR signaling (Crino, 2008). Mutations in genes within the mTOR pathway frequently cause focal cortical dysplasia (FCD), hemimegalencephaly, megalencephaly, and tuberous sclerosis complex (TSC). While mTORopathies may exemplify the role of cellular metabolism in the development of diseases related to uncontrolled cellular expansion, there are several MCD subtypes that may also inform how metabolic changes impact MCD pathogenesis. These MCD include polymicrogyria (PMG), lissencephaly, and certain cases of microcephaly.

## Cortical development and components of metabolism

In humans, the cortical preplate, a temporary precursor to the neocortex, forms at 7–8 weeks post conception (Terashima et al., 2021). Through a series of controlled spatiotemporal cell-fate decisions, new-born neurons migrate from the ventricular zone (VZ) along a radial glia (RG) scaffold to form the hexilaminar structure of the cerebral cortex. This migration of neurons into different cortical layers in an “inside-out” process produces the defined layers of the cortex and begins at 8 weeks post-conception with migration of neurons peaking at 18 weeks post-conception (Tau and Peterson, 2010; Govaert et al., 2020). Once positioned, neurons form synapses that are pruned as the brain develops and matures, and which continues into young-adulthood (Rodrigue et al., 2025). The high energy demand required for corticogenesis and lack of substantial energy reserves in the developed cortex necessitates the degradation of macromolecules to produce ATP that sustains both cortical development and post-development function (Aquilino et al., 2025). Supportive cells including astrocytes and oligodendrocytes also arise from neural stem cells at the VZ that are influenced by growth factors to become glial cells (Nutma et al., 2020). Astrocytes develop to become important metabolic regulators processing glucose, releasing lactate, and supplying neurons with glutamine among other processes (Pellerin and Magistretti, 1994; Costa et al., 2009). Oligodendrocytes, may not have a direct metabolic role but are key to CNS myelination, and thus expend a large amount of ATP during development (Kuhn et al., 2019).

It is important to note that there are differences in energy processing during development in humans compared to other mammals. For example, in mouse forebrain progenitor cells, glycolysis, beta-oxidation, and cholesterol metabolism occur at higher rates than in humans. Conversely, fatty acid synthesis occurs at higher rates in humans than rodents. Thus, there may be reduced translational potential of some rodent metabolic studies and further research will need to focus on model systems more aligned with human metabolic rates, e.g., resected human cortex or organoid models (Westi et al., 2022; Iwata et al., 2023). This will also need to be considered when developing therapeutics based on pre-clinical studies performed in rodent models.

## Macromolecules

### Glucose/carbohydrates

To produce a large amount of ATP, the brain must transport macromolecules including carbohydrates, lipids, and amino acids across the blood-brain barrier (BBB). Glucose is the obligate source of energy for the brain (Rho and Boison, 2022). Because developing neurons are dependent on the immediate availability of glucose, it must be transported into the brain or derived from endogenous glycogen stores in astrocytes (Pellerin and Magistretti, 1994; Régina et al., 2001). Several lines of evidence demonstrate that, for the purpose of ATP production during early corticogenesis, glucose is consumed through glycolysis, while in late corticogenesis, products of glycolysis and TCA are consumed via oxidative phosphorylation (OXPHOS) (Caravas and Wildman, 2014). In glycolysis, glucose is broken down into pyruvate producing a small amount of ATP while in OXPHOS, metabolized pyruvate products feed electrons through the electron transport chain to generate a proton gradient that makes a much larger amount of ATP. To facilitate these processes, glucose transporter 1 (GLUT1) moves more than 90% of glucose across the BBB (Ghosh et al., 2024).

During early cortical development, RG cells form the scaffolding upon which developing neurons migrate to form the cortical layers. RG reside in the ventricular zone, a transient embryonic layer that lines the ventricle, and have apical and basal processes that connect with cerebrospinal fluid (CSF). RG are self-renewing, dividing asymmetrically to give rise to early cortical neurons, while also dividing symmetrically to self-renew the RG population. Early cortical neurons can then climb and populate layers of the developing cortex along the RG scaffold. RGs generate all cortical excitatory neurons, astrocytes, and oligodendrocytes, and to do so are dependent on glucose metabolism and glycolysis (Andrews and Pearson, 2024). In mice, for example, moderate hyperglycemia results in an increased rate of neuronal differentiation from RG and early cell cycle exit. Conversely, in developing mice, severe hyperglycemia stalls neuronal differentiation and also extends the VZ (Rash et al., 2018). Further, self-renewing RG have a higher rate of glycolysis with elevated lactate production and lower OXPHOS, evidenced by high hypoxia-inducible factor 1-alpha (HIF-1α; a protein crucial to cellular response in hypoxic conditions) and the accumulation of glycogen in RG (Namba et al., 2021). Similarly, new-born pyramidal neurons go through rapid glycolysis in the presence of oxygen and produce lactate from pyruvate (Zheng et al., 2016). Then, as development progresses, neurons begin using OXPHOS to generate energy (Caravas and Wildman, 2014). As the demand for differentiated cortical neurons increases as corticogenesis proceeds, RG reduce production of lactate and lower their reliance on aerobic glycolysis. RG subsequently transition to a greater reliance on OXPHOS. This transition is highly sensitive and excess reactive oxygen species (ROS) can cause premature apoptosis and sustained neuronal immaturity. Interestingly, some amount of ROS in the environment is necessary to promote neuronal differentiation (Hollville et al., 2019; Rajan and Fame, 2024). Thus, a careful balance between glucose production, oxygen consumption, OXPHOS activity, and ROS production is necessary for neuronal differentiation and a sufficient radial glia population throughout corticogenesis. Disruption of this process (e.g., genetic mutations) could result in a sustained metabolic immaturity after development (similar to the immature neuronal phenotypes observed in MCD specimens) and could provide a potential target for therapy. key limiting step to glucose transport into the brain is transport across the BBB (see above). The integrity of the BBB is compromised by seizures, yet how this may affect transport of nutrients and neuronal metabolism remains unclear (Janigro and Walker, 2014; Marchi et al., 2014). A recent two-hit rat model of FCD discovered a Glut1-related deficit that gives insight into the role of metabolic dysregulation in the generation of seizures in FCD (Ghosh et al., 2022). Pregnant rats were irradiated at E17 to mimic the germline first-hit associated with FCD and after the pups were born, a second-hit was introduced by administering a dose of pentylenetetrazol (PTZ), a GABA receptor antagonist that mediates glutamate excitotoxicity and oxidative stress. The model captured many of the phenotypes associated with clinical cases of FCD such as mTOR hyperactivation, cortical disorganization, the presence of dysmorphic neurons, and epileptogenesis. Interestingly, Glut1 levels were reduced in rats with the irradiation first-hit, and further reduced in the cortices of rats that received the somatic second-hit, a phenomenon not previously captured by other FCD models (Wong, 2009). Additionally, normal Glut1 expression was found in the micro-vessels of the normal cortex of second-hit animals. Lactate levels were also assayed and showed a significant increase in both the one-hit and two-hit groups, compared to control an increase in energy consumption was also observed. Finally, BBB breakdown was evident in the *in utero*-irradiated rodent model during the pro-epileptogenic stage and was further weakened during epileptogenesis following a second-hit. These results emphasize the interconnected nature of mTOR dysfunction and nutrient acquisition. As glucose was depleted from the cortical environment, lactate levels increased in the dysplastic cortex. This drove neuronal hyperexcitability. While informative, the model itself may not be capturing the molecular genetic etiology of FCD. Indeed, the irradiation of pregnant rats with cesium-157 to model germline mutations may not recapitulate the region and cell-type specificity seen in FCD, and also likely has to-be-determined off-target effects (Nguyen and Bordey, 2022). Further, the somatic second-hit mutation delivered via PTZ administration does not accurately model how second-hits occur in VZ neural progenitor cells during corticogenesis. Indeed, PTZ is known to exaggerate seizure susceptibility globally, which doesn’t recapitulate the focal nature of FCD (Kajita and Mushiake, 2024). Further work using a transgenic or *in utero* electroporation (IUE)-based rodent model may be warranted.

**FIGURE 2:**
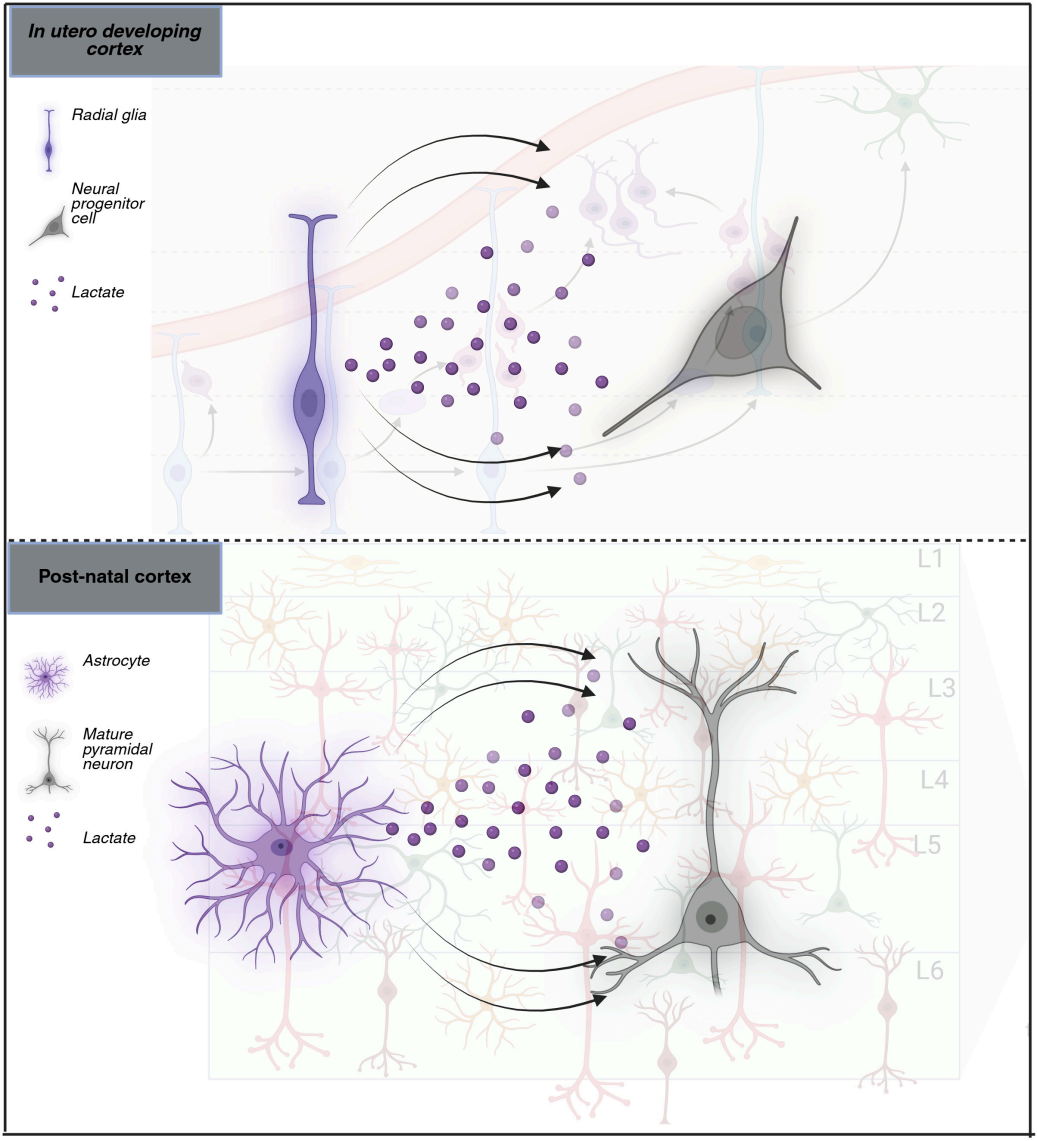
Astrocyte-neuron lactate shuttle. Schematic comparing the lactate shuttle between a mature pyramidal neuron and an astrocyte compared to a neural progenitor cell and a radial glia cell. Arrows denote lactate secretion from either the astrocyte or the radial glia, exemplifying a potential sustained depolarization of neurons throughout and after cortical development.

While it has yet to be examined in the context of epilepsy secondary to MCD, lactate metabolism is known to affect seizure initiation and propagation, as identified by *ex vivo* and *in vivo* models (Angamo et al., 2017; Skwarzynska et al., 2023). Furthermore, it is important to highlight the interconnected nature of astrocytic glutamate uptake, neuronal activation, excess energy production, and how that may support seizure development via lactate production, a molecular fuel related to carbohydrate metabolism. Astrocytes rapidly take up the amino acid glutamate released by depolarizing neurons at the postsynaptic cleft through glutamate transporters. Within astrocytes, glutamate is converted to glutamine, which is then transported back to neurons. When astrocytes take up glutamate, not only is there post-synaptic attenuation of nearby neurons, but within the astrocyte there is a switch from oxidative metabolism to aerobic glycolysis, and glucose is diverted from the TCA cycle to glycolysis and greater lactate production. This switch affects nearby neurons where the surge of lactate creates a rapid metabolic response in the neurons, with incoming lactate acting as an energy substrate, and depolarizing them to increase overall network excitability (Jha and Morrison, 2020). This hypothesis, the astrocyte-neuron lactate shuttle, remains a topic of debate (Figure 2). However, recent work supporting this hypothesis also demonstrated its role in the initiation and maintenance of seizures (Ksendzovsky et al., 2022). Interestingly, inhibition of lactate dehydrogenase, a metabolic pathway enzyme driving the neuronal depolarization, suppresses seizures *in vivo* in a mouse model of epilepsy (Pellerin and Magistretti, 1994; Sada et al., 2015; Chen and Zhu, 2025). Therefore, it is possible that during cortical development, when developing neurons transition from glycolysis to OXPHOS, some neurons do not go through the metabolic switch, resulting in neurons that remain in an undifferentiated state. Notably, while post-natally astrocytes are the main producers of lactate, the role is fulfilled during development by RG which express glycolytic enzymes, lactate dehydrogenase, and monocarboxylate transporters as early as embryonic day 10.5 (Dong et al., 2022). Thus, the immature neurons found in MCD subtypes may continue to function in the same manner, with developed astrocytes instead supplying them with the lactate they need to synchronously depolarize instead of RG.

A recent effort in mapping metabolic changes within the developing cortex has provided insight into vulnerable windows during brain development in which MCD can develop (Mil et al., 2025). Using both organoids and human primary cortical tissue, a recent study determined how low-nutrient environments impact corticogenesis. Specifically, extracellular acidification (a marker for glycolysis) and oxygen consumption rate (a marker for OXPHOS) were measure. In the human primary cortical tissue, there was an increase in OXPHOS during differentiation of neural progenitors into neurons, but there was no evident inverse relationship with the rate of glycolysis, which remained dynamic throughout development. For example, during upper-cortical layer neurogenesis, extracellular acidification remained high, demonstrating that glycolysis was active post-proliferation. This finding demonstrated that there may not be a discrete transitional timepoint from glycolysis to OXPHOS during cortical development, but it may exist on a continuum. Further, among the metabolites active across brain development, creatine abundance increased as development continued, while phosphocreatine decreased. This was consistent with the hypothesis that creatine is needed for energy-carrying when shifts in oxygen or glucose availability occur as cortex size increases. In low-glucose conditions, the cortical organoids had a significant reduction in glycolysis and activity of the pentose phosphate pathway (PPP), a key metabolic pathway for cell division and proliferation through its role in nucleotide synthesis (He et al., 2025). Transcriptomic analysis of cell-types in both human primary cortical tissue and cortical organoids treated with inhibitors of the PPP showed a change in cell fate made by cortical RG, and increased expression of genes associated with neuronal differentiation. Regardless of whether the PPP was inhibited indirectly through reduced glucose, or directly through pharmacologic inhibition or genetic knockdown, there was an increase in the number of outer RG, astrocytes, and inhibitory neurons.

The connection between PPP activity and cell-fate decisions raises several questions: (1) how do changes in metabolite abundance contribute to MCD? (2) and Do metabolically-linked the cell-fate choices made by early cortical neurons result in migration deficits and neuronal immaturity?, As both glycolysis and the PPP are critical for early neuronal cell-fate decisions, especially during low-glucose conditions (as is the case during early corticogenesis), the pathways could be highly active within MCD, promoting neurons within the lesion site to stay in an immature and undifferentiated state. Therefore, assaying the nature of glucose production and consumption in the MCD cortex, and whether pharmacological inhibition of glycolysis/PPP during early corticogenesis can affect the formation of MCD lesions without wholly disrupting development is an important next step.

### Lipids

Lipids are another source of energy for the CNS, and in the brain they are supplied by two processes: endogenous synthesis by glial cells and the transfer of free cholesterol across the BBB (Bruce et al., 2017). Most lipids cannot cross the BBB, but 20% of the energy demands by the brain are met through fatty acids oxidized in astrocytes to produce acetyl-CoA, NADH, and FADH for cellular respiration. Thus, it is essential for lipid reserves in the brain to be maintained (Yoon et al., 2022). Other than providing energy, brain lipids also aid in forming neurites, maintaining connectivity and cell signaling necessary for cognition, and sustaining the membranes of both neurons and glia (Luchtman and Song, 2013; Yoon et al., 2022). Raw materials such as fatty acids, phospholipids, and cholesterol are necessary for cellular biosynthesis during corticogenesis (Rajan and Fame, 2024). RG depend on the beta-oxidation of brain fatty acids, and the disruption of fatty-acid oxidation causes generation of two intermediate progenitor cell populations instead of the asymmetrical division usually seen with RG cells (Xie et al., 2016). Such a defect can lead to a dangerously small pool of RG, reducing the number and diversity of future neurons and glia (Subramanian et al., 2020).

In the brain there is a relatively low rate of oxidation of fatty acids due to the production of ROS- byproduct of cellular metabolism during mitochondrial OXPHOS that induces cell death in newly differentiated cortical neurons at higher concentrations (Schönfeld and Reiser, 2013). Interestingly, a recent lipidomic study of the developing brain showed that lipid metabolism is at its highest during cortical neurogenesis, a period of rapid division and preparation for migration, and not during synaptogenesis, the developmental process most-associated with high lipid metabolism (Bhaduri et al., 2021). Therefore, lipid metabolism may have different roles in cortical functioning *in utero* vs. post-natally, and in the case of MCDs, temporality of targeting lipid metabolism for therapeutics may be important.

Managing lipid metabolism has been a successful strategy to combat seizures and developmental delay that may result from MCD. Indeed, the ketogenic diet has shown efficacy in individuals living with epilepsy, with or without MCD. The ketogenic diet relies on a high-fat, low-carbohydrate intake of calories which helps drive the body into ketosis- a state which utilizes ketone bodies from fat to provide energy rather than through the breakdown of glucose. The breakdown of triglycerides in the intestine releases the fatty acids octanoic acid and decanoic acid, which can cross the BBB to provide energy for the brain (unlike most other fatty acids), and are a necessary energy source post-seizure (Sonnay et al., 2019). Interestingly, the ketogenic diet is known to reduce mTOR signaling (McDaniel et al., 2011). Fatty acids derived from ketosis reduce mTORC1 activity and subsequently reduce seizure frequency. Patients with malformations due to abnormal neural migration, and patients with malformations due to abnormal post-migration connectivity, responded positively to a ketogenic diet with a seizure reduction of more than 50% in 7 (31.8%) and 11 (64.7%) patients, respectively (Pasca et al., 2018).

Unfortunately, strict adherence to the ketogenic diet can be difficult due to differences in food accessibility and/or compliance issues. To address this, investigators directly administered decanoic acid, without change of diet (Warren et al., 2020). Using *D. discoideum* (a eukaryotic ameba with homologous genes to humans), they demonstrated that mTOR signaling is decreased by administration of decanoic acid in a UBX domain-containing protein (UBXD18; a protein that aids in amino acid homeostasis) dependent manner. Cell signaling moieties upstream of mTOR such as PI3K and AKT were unaffected. Further, in *D. discoideum*, decanoic acid decreased phosphorylated 4E-BP1 (a translation repressor directly phosphorylated by mTORC1) levels at 1 and 24 h. mTOR inhibition in these experiments were akin to starvation conditions- a known mechanism by which the mTOR pathway can be inhibited. In rat hippocampal slices, decanoic acid-dependent inhibition of mTORC1 signaling was observed in the absence of glucose deprivation or insulin signaling, both of which are associated with the ketogenic diet. Finally, astrocytes derived from patient iPSCs with *TSC1/TSC2* mutations, associated with dysregulated mTORC1 activity, were treated with decanoic acid. This treatment significantly decreased mTORC1 activation in both control and *TSC1* patient iPSCs. These results illustrate that the low compliance to the ketogenic diet and variable efficacy of mTOR-targeting drugs mean that some patients have a low rate of seizure cessation, and the targeted effect of decanoic acid may be in reducing mTOR activity. Furthermore, the ability of decanoic acid to reduce mTOR activity within astrocytes serves as an added benefit (Hochmuth and Hirrlinger, 2025). These findings may expand the type of therapeutics available for mTORopathies and other diseases shown to be worsened by mTOR pathway hyperactivity (Perluigi et al., 2015; Zou et al., 2020).

While modulating lipid metabolism has shown promise in some individuals with MCD and epilepsy, altering lipid metabolism in certain focal epilepsies may not allow for efficient utilization of brain lipids and may lead to increased neuronal excitability (Wang et al., 2024). For example, disruption of PI3K-mediated lipid signaling can underly focal epilepsy in patients with heterozygous variants in *PIK3C2B*- which encodes for the class II phosphatidylinositol 3-kinase PI3K-C2-beta, a ubiquitously expressed PI3K protein (Gozzelino et al., 2022). PIK3C2B is a key molecule in migratory, insulin, mTOR, and phosphatidylinositol synthesis signaling cascades. Screening for rare gene variants in focal epilepsy patients led to the identification of six *PIK3C2B* missense loss-of-function mutations, affecting either the catalytic or the lipid-binding domains of the lipid kinase. Generation of these variants in cell culture showed a reduced PI(3,4)P2-synthesizing ability, as well as impaired production of PI(3)P. The variant cells lines also had an increase in phosphorylated S6 protein- a marker for mTOR activity. Generation of *PI3KC2B* KO and heterozygous mice showed that the mutation did not cause any morphological aberrations in the affected neurons, nor did it result in any prominent cortical dyslamination. Electrophysiological studies of these mice revealed an increase in activity-dependent disinhibition of population spikes and marked polyspiking activity in more than half of the hippocampal slices from the KO mice and about 35% of the hippocampal slices from heterozygous mice. Patch-clamping the pyramidal neurons from the KO cohort showed that, upon electrical stimulation, there was a reduced number of inhibitory postsynaptic currents. These results are evidence that the ability to modulate neuronal excitability was weakened due to the variant, highlighted by a reduction in GABAergic feedback inhibition, and that the loss of the PI3KC2B kinase weakened inhibitory input. Taken together, these results demonstrate that alterations to lipid metabolism may be a source for focal epilepsy secondary to MCD and that the functional changes can occur independently of morphological changes (a hallmark of many MCD subtypes).

While the exact molecular mechanism by which defective lipid signaling can cause mTOR hyperactivity remains unidentified, the reduction in the formation of PI(3,4)P2 at the lysosome, where mTORC1 localizes, is hypothesized to be the mechanistic link. PI(3,4)P2 promotes autophagy and suppresses mTORC1 activity by stimulating retrograde movement of lysosomes toward the cell center—similar to what cells do during nutrient deprivation (Marat et al., 2017; Ebner et al., 2023). Thus, in MCD subtypes such as mTORopathies, the formation of PI(3,4)P2 may be suppressed and therefore may be a tractable therapeutic target.

### Amino acids

Amino acids play a critical role in neuronal growth and brain function. Disruption in amino acid metabolism can cause major changes in brain development and are therefore integral to understanding MCD pathogenesis. Like other macromolecules, many amino acids are transported across the BBB to be used in the brain (Miller et al., 1985; Knudsen et al., 1990). Glutamine is an amino acid that though it is locally abundant in the CNS, especially within astrocytes, also relies on specific amino-acid transporters to move across the BBB to ensure a consistent supply beyond reserves in astrocytes (Andersen and Schousboe, 2023).

In the brain, energy metabolism and neurotransmitter production and recycling are closely linked. For example, glutamine can undergo oxidation in the TCA cycle to produce ATP, alongside the neurotransmitters gamma-aminobutyric acid (GABA) and glutamate. Glutamine metabolism is also linked to glucose metabolism in the brain. After neurons release glutamate or GABA at the synaptic cleft, astrocytes scavenge neurotransmitters and metabolize them to form glutamine- a process that initiates glycolysis. Thus, glutamine cycling between neurons and glia not only highlights the interconnected nature of the two cell-types for metabolic processes but also how the differential processing of macromolecules is necessary to maximize available energy and cell-cell communication.

FCD is one of the most common MCD and is commonly caused by genetic variants in the subunits forming the GATOR1 complex- DEPDC5, NPRL3, and NPRL2. GATOR1 is the key subunit within the amino acid sensing arm of the mTOR pathway and therefore understanding how changes in neuronal amino acid metabolism impact brain development may provide important insights into FCD formation and pathogenesis. In brief, the amino acid sensing arm of the mTOR pathway detects the presence of intracellular amino acids through several amino acid sensing molecules that ultimately converge on the GATOR1 complex (Iffland et al., 2019; Fernandes and Demetriades, 2021). GATOR1 acts on the Rag GTPases which inhibits mTORC1 activity in low amino acid conditions. However, genetic variants in GATOR1 complex subunits cause persistent hyperactivation of mTORC1 regardless of ambient amino acid levels. Several amino acids are known to activate mTORC1 within *in vitro* models including alanine, arginine, asparagine, glutamine, histidine, leucine, methionine, serine, threonine, and valine (Takahara et al., 2020). Interestingly, glutamine can activate mTORC1 in a Rag-independent manner, but also activate mTORC1 indirectly by facilitating uptake of leucine, which the regulator can be exchanged with in order for activation to occur (Jewell et al., 2015).

Studying amino acid metabolism and the transport of amino acids in the brain has proven to be both biologically and technically challenging. Amino acids provide energy and serve as neurotransmitter precursors which results in both processes masking one another and confounding detailed analysis. Technical challenges include the transient nature of metabolites and contamination-prone technologies such as Liquid Chromatography-Mass Spectrometry (LC-MS) (Zhou et al., 2012; He and Wu, 2020). However, a recent study was able to define the neurometabolic abnormalities in subtypes of MCD including FCD, polymicrogyria (PMG), and gray matter heterotopia (GMH). Using proton magnetic resonance spectroscopy (1H-MRS), the lesion sites of 29 young adult patients were examined and metabolite measures for a host of amino acids, including glutamate and glutamine were acquired (Pu et al., 2023). In the PMG lesion sites, glutamine and glutamate were increased compared to the normal appearing contralateral side (NACS). In FCD, while there was no evidence of differences in glutamate and glutamine at the site, N-acetyl aspartate showed a significant reduction compared to the NACS. Findings in GMH lesions were similar to FCD.

These results have interesting implications. Different MCD may not behave similarly regarding which amino acids are being affected across mutations or MCD types, and it could be hypothesized that the timepoint in which these disorders are developing contribute to these differences. For example, GMH occurs secondary to migration arrest of neurons resulting in heterotopic neurons that also have reduced viability. Alternatively, neurons may be immature prior to migration, but retain the ability to migrate to atypical locations, which may be the case for dysmorphic neurons observed in FCD. Therefore, a necessary experiment would be to examine the directionality of the metabolic output through the tracking of cell viability from an early embryonic timepoint prior to neuron migration and layer formation. The increased presence of glutamine and glutamate in the PMG lesion site may be related to the later stage of cortical development in which PMG is believed to develop (when neurons have already migrated to their cortical layer) (Kolbjer et al., 2023). Glutamate/glutamine is necessary for excitatory neurotransmission and plays a major role in seizure initiation and spread, therefore their elevated presence at the PMG lesion site compared to the NACS may be a result of recurrent seizures and points toward defects in neuron-astrocyte glutamate cycling. Indeed, the formation of atypical excitatory circuit relies on the reuptake of glutamate in the form of glutamine by astrocytes, and excessive glutamate remaining at the synaptic cleft due to poor cycling can build circuitry that is prone to seizures and epilepsy (Chen et al., 2023; Gruenbaum et al., 2024). Taken together, these findings define well-known neurometabolites and their presence in MCD subtypes. However, mechanistic studies are warranted to isolate the specific conditions that contributed to these changes. Examining adult brains for metabolites that may lend to the etiology of a group of disorders known to form *in utero* does not necessarily provide a causal framework for the disorder.

Tuberous sclerosis complex is a rare multisystem disorder caused by genetic variants in *TSC1* or *TSC2.* A hallmark phenotype in TSC is the presence of cortical tubers- highly epileptogenic cortical lesions histologically defined by abnormal neuronal morphology and mTOR pathway hyperactivation within neurons (Feliciano, 2020). Recent studies point toward inflammation and differential uptake of amino acids as potential conduits for the epileptiform nature of tubers (Kagawa et al., 2005; Martin et al., 2017). A greater number of activated microglia and T-cells are found in tubers compared to surrounding NACS. Further, increased glial fibrillary acidic protein (GFAP) and reduced glutamine synthetase in resected human tubers, as well as reduced glutamate transporter expression in cortical astrocytes from mouse models of TSC raises the possibility that the metabolic processes necessary for preventing neurotoxicity in the cortex by glutamate conversion to glutamine is impaired (Boer et al., 2008; Zeng et al., 2010). Interestingly, the L-type amino acid transporter 1 (LAT1), is increased in cortical tubers (Lim et al., 2011). Increased expression of LAT1 is associated with active transport of large neutral amino acids into cells including leucine, phenylalanine, and tryptophan, which may point to certain amino acids being favored for metabolic processes in tubers over others. While glutamine is a critical fuel source for rapidly proliferating cells, large neutral amino acids are used primarily for protein synthesis directly downstream of mTOR activation. Interestingly, protein synthesis is decreased in some mouse models of TSC, even with hyperactive mTOR signaling (Saré et al., 2018). Therefore, it may be possible that amino acid transporters and their respective amino acids exist in a continuum of feedback loops which are dysregulated within TSC tubers, but whether that is a result of dyslamination of the cortex, altered proliferation, or reduced glial-neuron communication remains unknown.

## Mitochondrial metabolism and MCD

**FIGURE 3:**
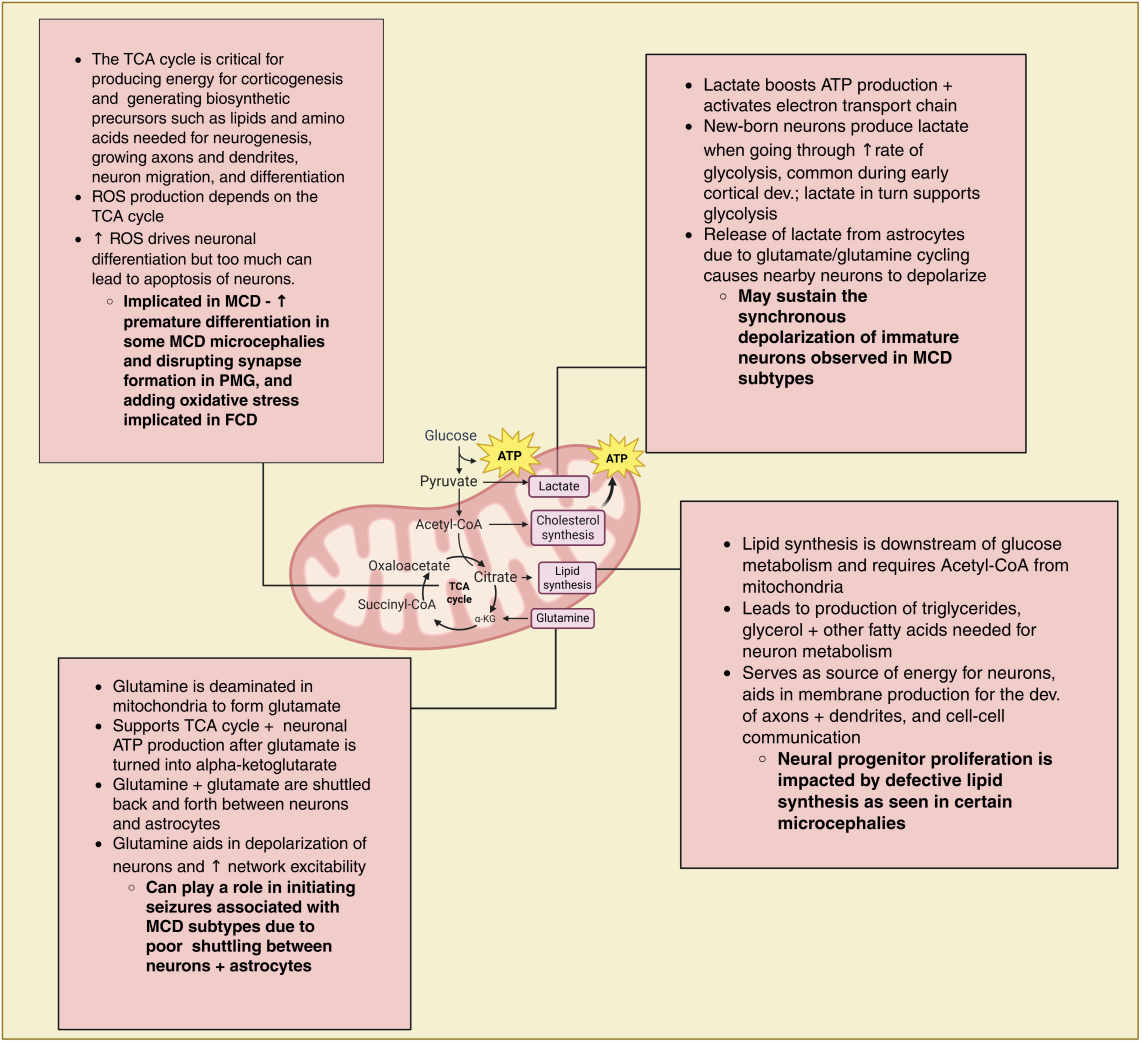
Mitochondrial metabolism and MCD. Overview of mitochondrial function and key findings in MCD associated with changes in mitochondrial function. Lines leading from boxes connect to the parts/processes of the mitochondrial that are impacted.

During cortical development mitochondria are central to many tightly coordinated metabolic processes that underlie the growth, proliferation, and differentiation of neurons. Mitochondria are vital for cellular respiration to meet the energy demands of growing cells, and perturbations in genes regulating and maintaining both mitochondrial function and morphology can have devastating effects on overall brain development and thus may play a role in MCD (Baum and Gama, 2021). Indeed, mitochondria play an important role in neuronal maturation, proliferation, circuit formation, and synaptogenesis- all processes impacted in MCD (Desikan and Barkovich, 2016; Fame and Lehtinen, 2021). Further, mitochondrial dynamics including fusion and fission play an important role in regulating metabolic processes linked to neuronal migration (Westermann, 2012). While the connection between mitochondrial dysfunction and MCD formation remains poorly understood (Figure 3) defective mitochondrial function has been observed in other disorders affecting cortical development (Fernandez et al., 2019; Buscaglia et al., 2020). The paucity of studies in this area is due, in part, to differences in cortical development and metabolic changes across model systems and limited access to *post-mortem* human embryonic tissue (Lodato and Arlotta, 2015). Despite this, there have been some landmark studies over the last two decades that show the effect of differences in mitochondrial structure and function on the synaptic activity of neurons (Fecher et al., 2019; Mandal and Drerup, 2019).

In the rare MCD and metabolic disorder Amish lethal microcephaly, cortical malformation occurs due to a mutation in solute carrier family 25 member 19 (*SLC25A19*). This gene encodes for a mitochondrial thiamine pyrophosphate carrier, which acts as a coenzyme inside the mitochondria for α-ketoglutarate dehydrogenase, a catalytic agent for the TCA cycle. The mutations leads to reduced OXPHOS, and results in seizures, developmental delays, and premature death, though the exact mechanism by which the variant causes disease is unclear (Ossola and Kalebic, 2022). In another case, a patient with a butyrobetaine gamma 2-oxoglutartate dioxygenase 1 (*BBOX1*) deletion also had microcephaly. *BBOX1* encodes for an enzyme that catalyzes the formation of L-carnitine, which supports the transportation of long-chain fatty acids into the mitochondria via the carnitine shuttle prior to oxidation and use in neural progenitor proliferation (Ferreira and McKenna, 2017). Further, patients with mitochondrial disorders sometimes exhibit cortical aberrations, though they are not considered MCD (Brown, 2005). For example, pyruvate dehydrogenase deficiency and fumarase deficiency result from mutations in a glycolytic enzyme and a TCA enzyme, respectively, and result in reduced neuronal glycolysis and OXPHOS. Patients with these deficiencies present with polymicrogyria, a thinning corpus collosum, severe developmental delays and seizures (Ottolenghi et al., 2011; Patel et al., 2012). While these disorders are not MCD, they highlight the role mitochondria may play in the cytoarchitecture of the human cerebral cortex. Unfortunately, there have been no cellular or molecular studies examining the mechanism by which dysfunctional mitochondria can cause neurons to under/over-migrate in MCD.

Abnormalities in oxidative metabolism have been implicated in other causes of epilepsy, even though it has not been explored in MCD. Temporal Lobe Epilepsy (TLE) is common type of epilepsy in which altered neuronal metabolism resulting from aberrant mitochondrial oxidative activity post-seizure and seizure-induced injury has been observed. There is strong evidence that deficits in mitochondrial oxygen consumption are found across various TLE mouse models. For example, a kainite-model of TLE showed that seizures increased the steady-state mitochondrial ROS levels in the forebrain, which caused deficits in mitochondrial respiration, exemplified by the significant reduction of oxygen consumption by hippocampal neurons (Rowley et al., 2015). Further, by examining hippocampal synaptosomes containing cytosolic and mitochondrial machinery during epileptogenesis, it has been observed that there is reduced ATP-linked baseline respiration of affected neurons during the acute and chronic phases of epileptogenesis. This suggests that damage to the electron transport chain along with a noticeable increase in ROS production, induces a greater susceptibility of animals to further seizures. Interestingly, during times when mitochondrial respiration was decreased, glycolytic rates remained unaltered, suggesting that glycolysis serves as the primary source of fuel during epileptogenesis (Gasior et al., 2010; Huynh et al., 2018). While these findings do not directly provide insight into the role of oxidative metabolism in MCD, they may provide insight into the presence of depolarizing dysmorphic neurons at MCD lesion sites. Indeed, if glycolysis is the main fuel source for seizures, and a switch from glycolysis to OXPHOS is necessary for proliferating new-born neurons to differentiate into mature neurons in the cortex, it is possible that dysregulation in the glycolysis/OXPHOS switch may be contributing to the pool of immature neurons that ultimately result in seizures in MCD.

## Discussion

The complex and elaborate nature of cortical development, during which a large amount of ATP is produced and utilized, necessitates a deeper understanding of metabolized macromolecules and the mitochondria. Cell metabolism is a multi-faceted and condition-sensitive process across organ systems, but the developing brain especially requires bioenergetic pathways that can be controlled in a continuous manner, rather than discrete “on” and “off” mechanisms that may sequester developing neurons into inappropriate niches and cortical positions. An emerging theme for neurodevelopmental disorders such as MCD, is that the disruption of metabolic regulation can drive pathological cellular phenotypes, and may integrate with signaling pathways such as mTOR, AMPK, and PI3K/AKT to sustain these phenotypes (He et al., 2025). These can include defective transport of macromolecules, atypical metabolite abundance, differential rates of glycolysis and/or OXPHOS, and defective mitochondrial functions such as an ineffective TCA cycle or carnitine shuttle.

**FIGURE 4:**
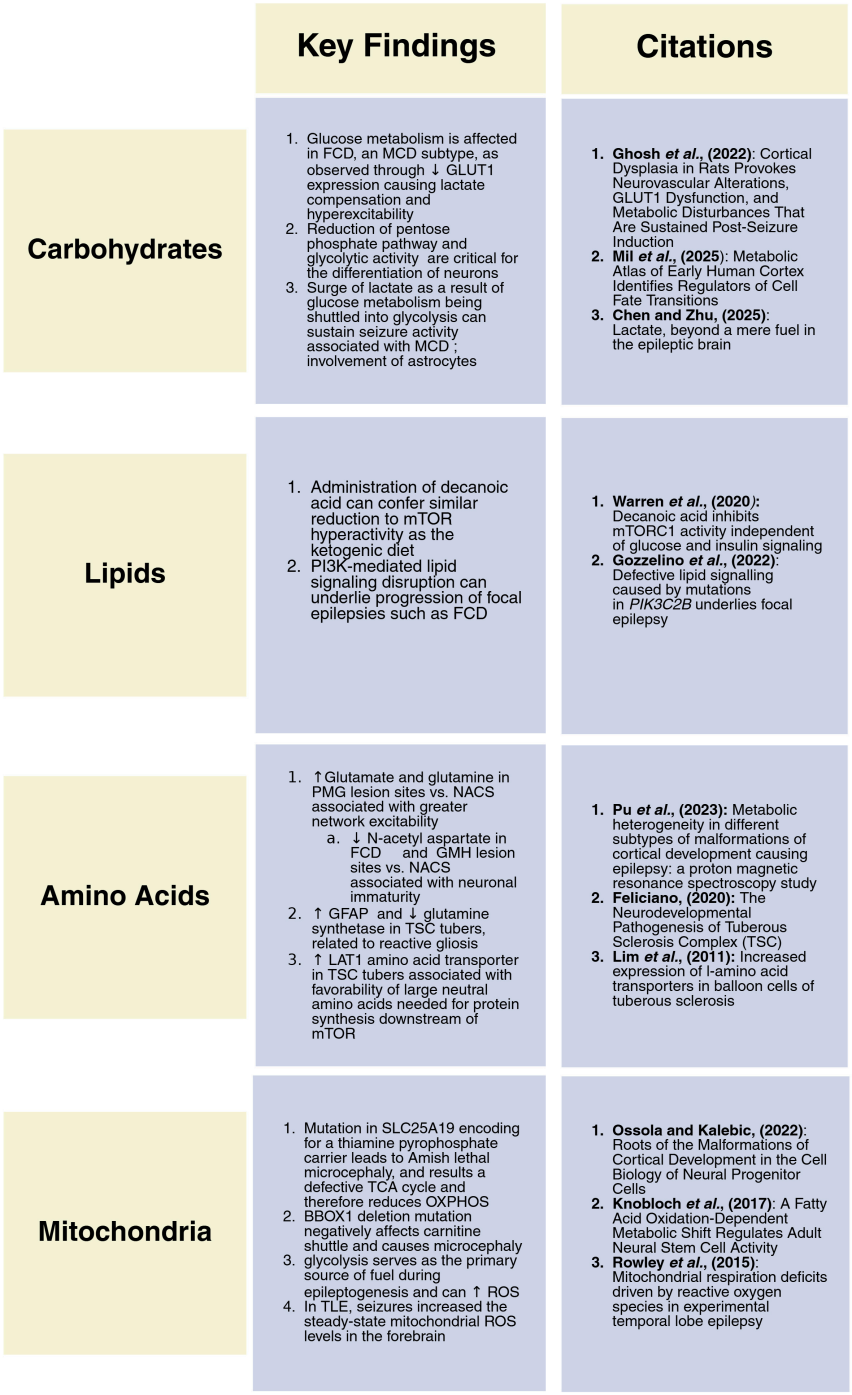
Summary of key findings and relevant citations. In each box we summarize the most relevant information related to how MCD impact each major macromolecule or the mitochondria and provide representative citations.

Macromolecules such as carbohydrates, lipids, and amino acids are crucial for providing energy substrates for cortical development (Figure 4). Changes in the production or metabolism of these molecules can affect the rate at which neurons develop, proliferate, and migrate to cortical layers ultimately impacting synaptogenesis. Downstream of these steps are abnormalities in circuit development that result in discrete cortical abnormalities impacting the function of the rest of the brain. As seen with certain MCD subtypes, neurons with defective macromolecule processing can depolarize together and cause seizures, but whether the metabolic processes are driving or sustaining them, remains unexplored.

Given that a unifying phenotype observed in various MCD subtypes is the presence of immature and dysmorphic neurons in incorrect layers, the state of the mitochondria within neurons should be further examine. Indeed, deficits within mitochondria could be the reason for differential ROS production, or an inefficient TCA cycle. Further, while early neurogenesis and cortical development is occurring in a relatively hypoxic environment, for a neuron to mature and differentiate mitochondria-based cellular respiration is crucial (Stepien and Wielockx, 2024). An interesting question that remains is whether in MCD is whether ROS production serves as an antagonistic or agonistic role in that process. Both hypoxia and ROS production are in flux during cortical development, similarly to glycolysis and oxidative respiration. Thus, it remains to be determined whether affecting this dynamic can serve as a therapeutic intervention. Indeed, addressing many of the unanswered questions posed in the paragraphs above will provide a basic framework upon which metabolic process could be leveraged for therapeutic potential to treat primary causes of MCD and the downstream consequences of epilepsy, ASD and ID.
